# Bayesian estimation for Dagum distribution based on progressive type I interval censoring

**DOI:** 10.1371/journal.pone.0252556

**Published:** 2021-06-02

**Authors:** Refah Alotaibi, Hoda Rezk, Sanku Dey, Hassan Okasha

**Affiliations:** 1 Mathematical Sciences Department, College of Science, Princess Nourah bint Abdulrahman University, Riyadh, Saudi Arabia; 2 Department of Statistics, Al-Azhar University, Cairo, Egypt; 3 Department of Statistics, St. Anthony’s College, Shillong, Meghalaya, India; 4 Department of Statistics, Faculty of Science, King AbdulAziz University, Jeddah, Saudi Arabia; Tongii University, CHINA

## Abstract

In this paper, we consider Dagum distribution which is capable of modeling various shapes of failure rates and aging criteria. Based on progressively type-I interval censoring data, we first obtain the maximum likelihood estimators and the approximate confidence intervals of the unknown parameters of the Dagum distribution. Next, we obtain the Bayes estimators of the parameters of Dagum distribution under the squared error loss (SEL) and balanced squared error loss (BSEL) functions using independent informative gamma and non informative uniform priors for both scale and two shape parameters. A Monte Carlo simulation study is performed to assess the performance of the proposed Bayes estimators with the maximum likelihood estimators. We also compute credible intervals and symmetric 100(1 − *τ*)% two-sided Bayes probability intervals under the respective approaches. Besides, based on observed samples, Bayes predictive estimates and intervals are obtained using one-and two-sample schemes. Simulation results reveal that the Bayes estimates based on SEL and BSEL performs better than maximum likelihood estimates in terms of bias and MSEs. Besides, credible intervals have smaller interval lengths than confidence interval. Further, predictive estimates based on SEL with informative prior performs better than non-informative prior for both one and two sample schemes. Further, the optimal censoring scheme has been suggested using a optimality criteria. Finally, we analyze a data set to illustrate the results derived.

## 1 Introduction

[1] introduced a heavy tail distribution, called the Dagum distribution, especially for modeling income distributions which could be used in place of log-normal and Pareto models. Since then, researchers have employed Dagum distribution for studying the distribution of wealth and income, reliability and survival data, etc. Further, Dagum distribution admits a mixture representation in terms of generalized gamma and inverse Weibull distributions. The distribution can also be obtained as a compound generalized gamma (GG) distribution whose scale parameter follows an inverse Weibull (IW) distribution with identical shape parameters. Lately, researchers have also adopted the Dagum distribution in the context of reliability and survival analysis (see [2–4]). [5, 6] carried out a detailed study on the origin and applications of the Dagum distribution. Recently, [7] studied the properties and different classical methods of estimation of the Dagum distribution.

A random variable *t* has the Dagum distribution with three parameters *θ*, *β* and λ if it has cumulative distribution function (CDF) (for *t* > 0) given by

$$\begin{array}{c} F\left(t;\theta ,\beta ,\lambda \right)={\left(1+\lambda t^{-\theta }\right)}^{-\beta }.\;\;\;\;\;t>0 \end{array}$$ (1)

The probability density function (pdf) corresponding to (1) reduces to

$$\begin{array}{c} f\left(t;\theta ,\beta ,\lambda \right)=\theta \beta \lambda t^{-\theta -1}{\left(1+\lambda t^{-\theta }\right)}^{-\beta -1},\;\;\;\;\;t>0 \end{array}$$ (2)

where *θ* and *β* are the shape parameters and λ is the scale parameter respectively. The reliability (survival) function is given by,

$$\begin{array}{c} R\left(t;\theta ,\beta ,\lambda \right)=1-{\left(1+\lambda t^{-\theta }\right)}^{-\beta },\;\;\;\;\;t>0 \end{array}$$ (3)

and hazard rate function is

$$\begin{array}{c} h\left(t;\theta ,\beta ,\lambda \right)=\frac{\theta \beta \lambda t^{-\theta -1}{\left(1+\lambda t^{-\theta }\right)}^{-\beta -1}}{1-{\left(1+\lambda t^{-\theta }\right)}^{-\beta }}.\;\;\;\;\;t>0 \end{array}$$ (4)

In several cases of life testing and survival analysis, the test unit is terminated before failure due to restriction of time, budget cost or accidental breakage. The data obtained from such cases may not be complete which is called a censored sample. For studying different observable physical phenomena, several censoring methodologies have been developed over the last several decades. In literature, type-I and type-II are the two most common censoring schemes which are widely used in reliability and life testing experiments (see [8]). However, none of these schemes allow the removal of any experimental units during the experiment. This limitations led to the development of progressive censoring scheme wherein test items are withdrawn before the termination of experiment (see [9] for more details). In many life testing experiments items put on a test are observed within an interval of time called interval censoring. However, this censoring schemes also does not allow removal of units in between the experiments. [10] introduced progressive type-I interval censoring scheme for the exponential distribution. In such type of censoring, items can be withdrawn between two prescheduled consecutive time points. In the recent past, progressive type-I interval censored scheme gained wide popularity due to its applicability in many practical problems.

In the recent past, several authors studied progressive type-I interval censored sampling schemes under varied conditions. In this regard, readers may refer to the works of [11–23].

The first objective of this paper is to obtain the unknown parameters of the model using the maximum likelihood estimators (MLEs). Further, we obtain the asymptotic confidence intervals for the unknown parameters. The second objective is to obtain Bayes estimators under SEL and BSEL functions using independent gamma priors for both scale and shape parameters of the model. We have also obtained Bayes credible intervals of the unknown parameters using Gibbs sampling technique. The third objective is to obtain Bayes predictive estimates and intervals based on the observed samples using one-sample and two-sample schemes. Finally, the fourth objective is to obtain optimal censoring schemes. As far as we know, no study has attempted to study three parameter distribution by taking into account one and two-sample prediction intervals in the Bayesian framework along with optimal censoring schemes using progressively type-I interval censored schemes perhaps, due to complexities in computational work.

The remainder of this article is organized in the following manner: In Section 3, a brief discussion on progressive type-I interval censored sampling is presented. Section 4 deals with maximum likelihood estimation and its associated approximate confidence intervals. Section 5 discusses Bayesian estimation and its credible intervals. Section 6 includes a discussion on Bayesian prediction based on one sample and two sample schemes. A simulation study is conducted in Section 7, to compare the performance of various estimates developed in this paper. In Section 8 deals with optimal censoring schemes. In Section 9, with the help of a real data set estimation procedures developed in the previous sections are illustrated. Finally, conclusion is presented in Section 10.

## 2 Progressive type-I interval censoring

Assume that *n* identical items are put on a life test at time *t*_0_ = 0 and under inspection at pre-specified times 0 < *t*_1_ < *t*_2_ < … < *t*_*m*_ where *t*_*m*_ is the schedule time to terminate the experiment and *m* is prefixed. At time *t*_*j*_, the total number of observed failures in the interval (*t*_*j*−1_ − *t*_*j*_] are *D*_*j*_. Further, assume that *R*_*j*_ alive items are removed randomly from the life testing at time *t*_*j*_, *j* = 1, 2, …, *m*. Here the number of surviving units at time *t*_*j*_, say *S*_*j*_ is a random variable, and therefore *R*_*j*_ ≤ *S*_*j*_. Thus, *R*_*j*_ could be determined by the pre-fixed percentage values *p*_1_, *p*_2_, …, *p*_*m*−1_ with *p*_*m*_ = 1, *R*_*j*_ be determined by *R*_*j*_ = [*S*_*j*_ *p*_*j*_], for *j* = 1, 2, …, *m* − 1. So the data in progressive type-I interval censoring is given by *T*_*j*_ = (*D*_*j*_, *R*_*j*_, *t*_*j*_), *j* = 1, 2, …, *m*. If *R*_*j*_ = 0, for *j* = 1, 2, …, *m* − 1, and $R_{m}=n-\sum_{j=1}^{m}D_{j}$, then progressive type I interval censoring scheme reduces to the normal interval censored sample.

## 3 Maximum likelihood estimators

Given the progressively type-I censored data, *T*_*j*_ = (*D*_*j*_, *R*_*j*_, *t*_*j*_), *j* = 1, 2, …, *m* of size *n*, from a continuous lifetime distribution with CDF as defined in (1), then the likelihood function can be written as ([10])

$$\begin{array}{c} L\left(data|\phi \right)\propto \prod_{j=1}^{m}{\left(F\left(T_{j};\phi \right)-F\left(T_{j-1};\phi \right)\right)}^{D_{j}}{\left(1-F\left(T_{j};\phi \right)\right)}^{R_{j}}, \end{array}$$ (5)

where *t*_0_ = 0 and *ϕ* ≡ (*θ*, *β*, λ) is the parameter vector.. For the CDF of Dagum distribution defined in (1), the likelihood function (5) can be specified as follows:

$$\begin{array}{c} L\left(data|\phi \right)\propto \prod_{j=1}^{m}{\left({\left(1+\lambda \left(t_{j}^{-\theta }\right)\right)}^{-\beta }-{\left(1+\lambda {\left(t_{j-1}\right)}^{-\theta }\right)}^{-\beta }\right)}^{D_{j}}{\left(1-{\left(1+\lambda {\left(t_{j}\right)}^{-\theta }\right)}^{-\beta }\right)}^{R_{j}}. \end{array}$$ (6)

The log likelihood function, after ignoring the constant of proportionality, is given by

$$\begin{array}{c} l\left(data|\phi \right)\propto \sum_{j=1}^{m}[D_{j}log\left({\left(1+\lambda t_{j}^{-\theta }\right)}^{-\beta }-{\left(1+\lambda t_{j-1}^{-\theta }\right)}^{-\beta }\right)+R_{j}log\left(1-{\left(1+\lambda {\left(t_{j}\right)}^{-\theta }\right)}^{-\beta }\right)]. \end{array}$$ (7)

The corresponding normal equations are

$$\begin{array}{ccc} \frac{\partial l(data|\phi )}{\partial \theta } & = & \sum_{j=1}^{m}(\frac{D_{j}{\left(U_{j}\right)}_{\theta }}{U_{j}}-\frac{\beta \lambda R_{j}\log(t_{j})}{V_{j}})=0, \end{array}$$ (8)

$$\begin{array}{ccc} \frac{\partial l(data|\phi )}{\partial \beta } & = & \sum_{j=1}^{m}(\frac{D_{j}{\left(U_{j}\right)}_{\beta }}{U_{j}}+\frac{R_{j}\log(\lambda t_{j}^{-\theta }+1)}{(\lambda t_{j}^{-\theta }+1){}^{\beta }-1})=0, \end{array}$$ (9)

$$\begin{array}{ccc} \frac{\partial l(data|\phi )}{\partial \lambda } & = & \sum_{j=1}^{m}(\frac{D_{j}{\left(U_{j}\right)}_{\lambda }}{U_{j}}+\beta R_{j}V_{j})=0, \end{array}$$ (10)

where

$$\begin{array}{ccc} U_{j}\equiv U_{j}(\theta ,\beta ,\lambda ) & = & (\lambda t_{j}^{-\theta }+1){}^{-\beta }-(\lambda t_{j-1}^{-\theta }+1){}^{-\beta }, \end{array}$$ (11)

$$\begin{array}{ccc} V_{j}\equiv V_{j}(\theta ,\beta ,\lambda ) & = & (t_{j}^{\theta }+\lambda )((\lambda t_{j}^{-\theta }+1){}^{\beta }-1), \end{array}$$ (12)

$$\begin{array}{c} {\left(U_{j}\right)}_{\theta }\equiv \frac{\partial U_{j}(\theta ,\beta ,\lambda )}{\partial \theta }=t_{j}^{-\theta }\log(t_{j}){\left(\lambda t_{j}^{-\theta }+1\right)}^{-\beta -1}-t_{j-1}^{-\theta }\log(t_{j-1})(\lambda t_{j-1}^{-\theta }+1){}^{-\beta -1}, \end{array}$$ (13)

$$\begin{array}{c} {\left(U_{j}\right)}_{\beta }\equiv \frac{\partial U_{j}(\theta ,\beta ,\lambda )}{\partial \beta }=(\lambda t_{j-1}^{-\theta }+1){}^{-\beta }\log(\lambda t_{j-1}^{-\theta }+1)-(\lambda t_{j}^{-\theta }+1){}^{-\beta }\log(\lambda t_{j}^{-\theta }+1), \end{array}$$ (14)

and

$$\begin{array}{c} {\left(U_{j}\right)}_{\lambda }\equiv \frac{\partial U_{j}(\theta ,\beta ,\lambda )}{\partial \lambda }=t_{j-1}^{-\theta }(\lambda t_{j-1}^{-\theta }+1){}^{-\beta -1}-t_{j}^{-\theta }(\lambda t_{j}^{-\theta }+1){}^{-\beta -1}. \end{array}$$ (15)

The maximum likelihood estimates ${\hat{\theta }}_{MSL}$, ${\hat{\beta }}_{MSL}$ and ${\hat{\lambda }}_{MSL}$ of *θ*, *β* and λ, respectively, can be obtained by solving the Eqs (8), (9) and (10), respectively. For more details about the maximum likelihood estimates see for example Dong et al. [24], Chen et al. [25] and Chen et al. [26]. For interval estimation of the model parameters, we require the observed information matrix. Applying the usual large sample approximation, asymptotic distribution of the MLE $\hat{\phi }$ is $\left(\hat{\phi }-\phi \right)\to N\left(0,I^{-1}\left(\phi \right)\right)$, see [27], where *I*(*ϕ*), the expected Fisher information matrix of the unknown parameters *ϕ* = (*θ*, *β*, λ) is

$$\begin{array}{c} I\left(\phi \right)=-E[\begin{array}{ccc} \frac{\partial^{2}l}{\partial \theta^{2}} & \frac{\partial^{2}l}{\partial \theta \partial \beta } & \frac{\partial^{2}l}{\partial \theta \partial \lambda }\\ \frac{\partial^{2}l}{\partial \beta \partial \theta } & \frac{\partial^{2}l}{\partial \beta^{2}} & \frac{\partial^{2}l}{\partial \beta \partial \lambda }\\ \frac{\partial^{2}l}{\partial \lambda \partial \theta } & \frac{\partial^{2}l}{\partial \lambda \partial \beta } & \frac{\partial^{2}l}{\partial \lambda^{2}} \end{array}]=-E[\begin{array}{ccc} \Psi_{\theta \theta } & \Psi_{\theta \beta } & \Psi_{\theta \lambda }\\ \Psi_{\beta \theta } & \Psi_{\beta \beta } & \Psi_{\beta \lambda }\\ \Psi_{\lambda \theta } & \Psi_{\lambda \beta } & \Psi_{\lambda \lambda } \end{array}], \end{array}$$ (16)

and *I*^−1^(*ϕ*), the 3 × 3 inverse of the observed information matrix of the unknown parameters *ϕ* = (*θ*, *β*, λ) is

$$\begin{array}{c} I^{-1}\left(\phi \right)=-E{\left[\begin{array}{ccc} \Psi_{\theta \theta } & \Psi_{\theta \beta } & \Psi_{\theta \lambda }\\ \Psi_{\beta \theta } & \Psi_{\beta \beta } & \Psi_{\beta \lambda }\\ \Psi_{\lambda \theta } & \Psi_{\lambda \beta } & \Psi_{\lambda \lambda } \end{array}\right]}_{\theta =\hat{\theta },\beta =\hat{\beta },\lambda =\hat{\lambda }}^{-1}={\left[\begin{array}{ccc} \text{var}(\theta ) & \text{cov}(\theta ,\beta ) & \text{cov}(\theta ,\lambda )\\ \text{cov}(\beta ,\theta ) & \text{var}(\beta ) & \text{cov}(\beta ,\lambda )\\ \text{cov}(\lambda ,\theta ) & \text{cov}(\lambda ,\beta ) & \text{var}(\lambda ) \end{array}\right]}_{\theta =\hat{\theta },\beta =\hat{\beta },\lambda =\hat{\lambda }}. \end{array}$$ (17)

A 100(1 − *τ*)% approximate confidence intervals for each parametercan be obtained as follows: $\hat{\theta }\pm Z_{\frac{\tau }{2}}\sqrt{\text{var}(\hat{\theta })}$, $\hat{\beta }\pm Z_{\frac{\tau }{2}}\sqrt{\text{var}(\hat{\beta })},$ and $\hat{\lambda }\pm Z_{\frac{\tau }{2}}\sqrt{\text{var}(\hat{\lambda })},$ where $Z_{\frac{\tau }{2}}$ is the upper ${\left(\frac{\tau }{2}\right)}^{th}$ percentile of the standard normal distribution.

## 4 Bayesian analysis

This section considers Bayes estimation of the parameters. When all the parameters are unknown, no jooint conjugate prior is available. In such a situation, there are number of ways to choose the priors. Here, we consider the piecewise independent gamma priors [see [28–31]]. It is assumed that *θ*, *β* and λ have the independent gamma prior distributions with pdf given by

$$\begin{array}{c} g\left(\theta \right)\propto \theta^{a_{1}-1}e^{-b_{1}\theta }\;\;\;\theta >0 \end{array}$$ (18)

$$\begin{array}{c} g\left(\beta \right)\propto \beta^{a_{2}-1}e^{-b_{2}\beta }\;\;\;\beta >0 \end{array}$$ (19)

$$\begin{array}{c} g\left(\lambda \right)\propto \lambda^{a_{3}-1}e^{-b_{3}\lambda }\;\;\;\lambda >0 \end{array}$$ (20)

Here all the hyper parameters *a*_1_, *b*_1_, *a*_2_, *b*_2_, *a*_3_ and *b*_3_ are assumed to be known. Thus, the joint prior distribution is given by

$$\begin{array}{c} g\left(\phi \right)\propto \theta^{a_{1}-1}\beta^{a_{2}-1}\lambda^{a_{3}-1}e^{-(b_{1}\theta +b_{2}\beta +b_{3}\lambda )},\;\;\;\phi >0 \end{array}$$ (21)

Combining (21) with (6), the joint posterior distribution is derived as

$$\begin{array}{ccc} \pi (\phi |\underline{t}) & = & g(\phi )L(data|\phi ) \end{array}$$ (22)

The marginal posterior probability density functions of *θ*, *β* and λ are given respectively as

$$\begin{array}{cc} \pi_{1}\left(\theta |\beta ,\lambda ,\underline{t}\right) & =\int_{0}^{\infty }\int_{0}^{\infty }g\left(\phi \right)L\left(data|\phi \right)\;d\beta \;d\lambda , \end{array}$$ (23)

$$\begin{array}{cc} \pi_{2}\left(\beta |\theta ,\lambda ,\underline{t}\right) & =\int_{0}^{\infty }\int_{0}^{\infty }g\left(\phi \right)L\left(data\phi \right)\;d\theta \;d\lambda , \end{array}$$ (24)

$$\begin{array}{cc} \pi_{3}\left(\lambda |\theta ,\beta ,\underline{t}\right) & =\int_{0}^{\infty }\int_{0}^{\infty }g\left(\phi \right)L\left(data|\phi \right)\;d\theta \;d\beta . \end{array}$$ (25)

### 4.1 Bayes estimators under squared error loss function

Bayes estimator of the parameters under SEL function are nothing but the posterior mean of the corresponding parameters. Hence the Bayes estimator ${\hat{\theta }}_{SEL}$ of *θ* under SEL can be simply expressed as

$${\hat{\theta }}_{SEL}=E\left(\theta |\underline{t}\right)=\int_{0}^{\infty }\;\theta \;\pi_{1}\left(\theta |\beta ,\lambda ,\underline{t}\right)\;d\theta$$

In a similar way, we can obtain the estimators ${\hat{\beta }}_{SEL}$ and ${\hat{\lambda }}_{SEL}$ of *β* and λ, respectively, as given below:

$${\hat{\beta }}_{SEL}=E\left(\beta |\underline{t}\right)=\int_{0}^{\infty }\;\beta \;\pi_{2}\left(\beta |\theta ,\lambda ,\underline{t}\right)\;d\beta ,$$

and

$${\hat{\lambda }}_{SEL}=E\left(\lambda |\underline{t}\right)=\int_{0}^{\infty }\;\lambda \;\pi_{3}\left(\lambda |\theta ,\beta ,\underline{t}\right)\;d\lambda .$$

As these estimators can not be obtained explicitly, so the estimates are obtained by numerical method.

### 4.2 Bayes estimators based on balanced squared error loss function

Instead of using the well known symmetric SEL function, one can use balanced squared error loss function (BSEL) which was first proposed by [32] and subsequently extended class of BSEL function was introduced by [33] and can be expressed as:

$$\begin{array}{c} L\left(\phi ,\hat{\phi }\right)=\omega L\left({\hat{\phi }}_{0},\phi \right)+\left(1-\omega \right)L\left(\phi -\hat{\phi }\right), \end{array}$$ (26)

where $L(\phi ,\hat{\phi })$ is an arbitrary loss function, when ${\hat{\phi }}_{0}$ is a chosen estimator of *ϕ* and the weight *ω* ∈ [0, 1]. The BSEL is a generalized loss functions which includes absolute error loss function, entropy loss function, LINEX loss function and generalizes SEL function.

[34] suggested the use of BSEL function, if $L\left(\phi ,\hat{\phi }\right)={\left(\hat{\phi }-\phi \right)}^{2}$ is substituted in (26), the BSEL function can be obtained and given by the following form:

$$\begin{array}{c} L_{BSEL}\left(\phi ,\hat{\phi }\right)=\omega {\left(\phi -\hat{\phi_{0}}\right)}^{2}+\left(1-\omega \right){\left(\phi -\phi^{*}\right)}^{2}, \end{array}$$ (27)

the corresponding Bayes estimator ${\hat{\phi }}_{BSEL}$ of a function *ϕ* using *BSEL* is given by

$$\begin{array}{c} {\hat{\phi }}_{BSEL}=\omega {\hat{\phi }}_{ML}+\left(1-\omega \right){\hat{\phi }}_{SEL}. \end{array}$$ (28)

where ${\hat{\phi }}_{ML}$ is the MLE of *ϕ* and ${\hat{\phi }}_{SEL}$ of *ϕ* based on SEL function, where *ϕ* = (*θ*, *β*, λ).

### 4.3 Credible intervals

The Bayesian interval estimates can be derived more directly than the frequentist confidence interval estimates. After obtaining the marginal posterior distribution of *ϕ*, a symmetric 100(1 − *τ*)% two-sided Bayes probability interval estimate of *ϕ*, denoted by [*L*_*ϕ*_, *U*_*ϕ*_], can be obtained by solving the following equation. The Bayesian analog to the confidence interval is called a credible interval. In general,

$$\begin{array}{c} p\left[L\left(\underline{t}\right)<\phi <U\left(\underline{t}\right)\right]=\int_{L(\underline{t})}^{U(\underline{t})}\pi \left(\Theta |\underline{t}\right)\;d\phi =1-\tau \end{array}$$ (29)

for the limits *L*_*ϕ*_ and *U*_*ϕ*_. We need to apply suitable numerical method to compute the above intervals.

## 5 Prediction of future values

In the following two subsections, we will investigate the one and two-sample prediction intervals in the Bayesian framework.

### 5.1 One-sample prediction

[35] obtained 100(1 − *τ*)% predictive intervals based on the two sample schemes. Let *t*_1_ < *t*_2_ < … < *t*_*r*_ be the ordered informative sample from a distribution function whose CDF is *F*(*x*).

#### 5.1.1 Point estimation

The future sample consists of the remaining order statistics *t*_*r*+1_ < *t*_*r*+2_ < … < *t*_*n*_.

Let *Y*_*s*_ = *t*_*r*+*s*_, *s* = 1, 2, …, *n* − *r*. Assume *f*_*s*_(*y*_*s*_|*ϕ*) to denote the pdf of the *s*^*th*^ unit to fail, given that the *r*^*th*^ unit had already failed.

$$\begin{array}{c} f_{s}\left(y_{s}|\phi \right)\propto {\left[F\left(y_{s}|\phi \right)-F\left(t_{r}|\phi \right)\right]}^{s-1}{\left[1-F\left(y_{s}|\phi \right)\right]}^{n-r-s}{\left[1-F\left(t_{r}|\phi \right)\right]}^{-(n-r)}f\left(y_{s}|\phi \right), \end{array}$$ (30)

The binomial expansion of each of the first two terms on the right-hand side then yields

$$\begin{array}{c} f_{s}\left(y_{s}|\phi \right)\propto \sum_{j_{1}=0}^{s-1}\sum_{j_{2}=0}^{n-r-s}D_{j_{1}}D_{j_{2}}{\left[F\left(t_{r}|\phi \right)\right]}^{j_{1}}{\left[F\left(y_{s}|\phi \right)\right]}^{s-1-(j1-j2)}{\left[1-F\left(t_{r}|\phi \right)\right]}^{-(n-r)}f_{s}\left(y_{s}|\phi \right), \end{array}$$ (31)

where *F*(.|*ϕ*) and *f*(.|*ϕ*) are given by (1) and (2), respectively, *ϕ* = (*θ*, *β*, λ), Dj1=(-1)j1(s-1j1), and Dj2=(-1)j2(n-r-sj2). Therefore, (31) becomes

$$\begin{array}{c} \begin{array}{cc} f_{s}\left(y_{s}|\phi \right) & \propto \sum_{j_{1}=0}^{s-1}\sum_{j_{2}=0}^{n-r-s}D_{j_{1}}D_{j_{2}}\theta \beta \lambda y_{s}^{-\theta -1}{\left(1+\lambda y_{s}^{-\theta }\right)}^{-\beta (s-j_{1}+j_{2})-1}{\left(1+\lambda t_{r}^{-\theta }\right)}^{-\beta j_{1}}\\ & \times {\left(1-(1+\lambda t_{r}^{-\theta }){)}^{-\beta }\right)}^{-(n-r)}. \end{array} \end{array}$$ (32)

The predictive density function of the future order statistic *y*_*s*_, *s* = 1, 2, …, *n* − *r*, is given by

$$\begin{array}{ccc} f_{s}^{*}\left(y_{s}|\underline{t}\right) & = & \int_{\phi }f_{s}\left(y_{s}|\phi \right)\pi \left(\phi |\underline{t}\right)\;d\phi ,\;\;\;\;\;\;\;\;\;\;y_{s}>t_{r} \end{array}$$ (33)

$$=\int_{\phi }\sum_{j_{1}=0}^{s-1}\sum_{j_{2}=0}^{n-r-s}D_{j_{1}}D_{j_{2}}\theta \beta \lambda y_{s}^{-\theta -1}{\left(1+\lambda y_{s}^{-\theta }\right)}^{-\beta (s-j_{1}+j_{2})-1}{\left(1+\lambda t_{r}^{-\theta }\right)}^{-\beta j_{1}}\times {\left(1-(1+\lambda t_{r}^{-\theta }){)}^{-\beta }\right)}^{-(n-r)}\pi \left(\phi |\underline{t}\right)\;d\phi ,$$ (34)

where $\pi (\phi |\underline{t})$ is as given by (22).

#### 5.1.2 Prediction bounds

In general $\left(L\left(\underline{t}\right)<Y_{\left(s\right)}<U\left(\underline{t}\right)\right)$ is a 100(1 − *τ*)% credibility interval for *Y*_(*s*)_ if

$$\begin{array}{c} p(L\left(\underline{t}\right)<Y_{\left(s\right)}<U\left(\underline{t}\right))=\int_{L(\underline{t})}^{U(\underline{t})}f_{s}^{*}\left(y_{s}|\underline{t}\right)\;dy_{\left(s\right)}=1-\tau , \end{array}$$

Therefore, we can obtain the Bayesian predictive bounds for *Y*_(*s*)_ as:

$$\begin{array}{c} p(Y_{\left(s\right)}\geq q_{s}|\underline{x})=\int_{q_{s}}^{\infty }f_{s}^{*}\left(y_{s}|\underline{t}\right)\;dy_{\left(s\right)}. \end{array}$$

where $f_{s}^{*}\left(y_{s}|\underline{t}\right)$ is given in (34)

### 5.2 Two samples prediction

Assume that {*Y*_1_, …, *Y*_*m*_} be the future sample of size *m* independent of the informative sample {*T*_1_, …, *T*_*m*_}. Let *Y*_(1)_ < … < *Y*_(*m*)_ be the order statistics (OS) of the future sample. In order to obtain the predictive density of the OS *Y*_(*r*)_ of the future sample given the informative sample {*T*_1_, …, *T*_*m*_}, the probability density function of the *s*^*th*^ OS of the future sample is given by

$$\begin{array}{c} f_{r}\left(y_{r}|\phi \right)=\frac{m!}{(r-1)!(m-r)!}{\left[F\left(y_{r}|\phi \right)\right]}^{r-1}{\left[1-F\left(y_{r}|\phi \right)\right]}^{m-r}f\left(y_{r}|\phi \right), \end{array}$$ (35)

where *f*(.|*ϕ*) is as given in (2) and *F*(.|*ϕ*) denotes the corresponding CDF of *f*(.|*ϕ*). Let $f_{r}^{*}\left(.|\underline{t}\right)$ be the predictive density of *Y*_(*r*)_, then

$$\begin{array}{c} f_{r}^{*}\left(y_{r}|\underline{t}\right)=\int_{\phi }f_{r}\left(y_{r}|\phi \right)\pi \left(\Theta |\underline{t}\right)\;d\phi . \end{array}$$ (36)

where $\pi (\Theta |\underline{t})$ is defined in (22). Since, $f_{r}^{*}\left(y_{r}|\underline{t}\right)$ cannot be expressed in closed form, it cannot be evaluated analytically. A symmetric 100 *γ*% predictive interval for *Y*_(*r*)_ for the lower bound *L* and upper bound *U* can be obtained by solving the following non-linear Eqs (37) and (38).

$$\begin{array}{c} p\left(Y_{\left(r\right)}\leq L|\underline{t}\right)=\frac{1}{2}-\frac{\gamma }{2}, \end{array}$$ (37)

$$\begin{array}{c} p\left(Y_{\left(r\right)}\leq U|\underline{t}\right)=\frac{1}{2}+\frac{\gamma }{2}. \end{array}$$ (38)

Here we need to apply suitable numerical method to compute the above intervals.

## 6 Simulation study

In this section, we obtain the maximum likelihood estimates (MLE), the Bayes estimates of the unknown parameters of the Dagum distribution under SEL and BSEL functions through a simulation study using R language. We obtain one and two-sample Bayes predictive estimates based on observed samples. The MLE of the model parameters *ϕ* and the approximate 95% confidence intervals are obtained using progressively type-I interval censored data. Here, we consider two different values of *n* such as *n* = 50, 100. In Table 2, we report the mean square error (MSE) values for the maximum likelihood estimators of the Dagum distribution model parameters *ϕ*. We have also considered Bayesian approach of estimating the Dagum distribution parameters *ϕ* by assuming independent gamma distribution prior, *θ* ∼ *gamma*(*a*_1_, *b*_1_), *β* ∼ *gamma*(*a*_2_, *b*_2_) and λ ∼ *gamma*(*a*_3_, *b*_3_) with known and non-negative values for the hyper parameters *a*_1_, *a*_2_, *a*_3_, *b*_1_, *b*_2_ and *b*_3_ using the SEL and BSEL functions and for both informative and non-informative priors. We generate the progressive type I interval censored sampling data, *H*_*j*_ = {*D*_*j*_, *R*_*j*_, *t*_*j*_} of the Dagum distribution by first generating *n* random variables *U*_1_, *U*_2_, …, *U*_*n*_, *n* ≥ *m* from a *U*(0, 1) distribution. The data *t*_1_, *t*_2_, …, *t*_*n*_ are there by calculated using $t_{j}=\frac{1}{\lambda }{\left(U_{j}^{\frac{-1}{\beta }}-1\right)}^{\frac{-1}{\theta }}$ where the number *D*_*i*_ of failures within (*t*_*i*−1_, *t*_*i*_] are generated and *R*_*i*_ surviving items are randomly removed from the testing based on the pre-specified inspection times *t*_1_ ≤ … ≤ *t*_*m*_ and the pre-specified percentage *p* = (*p*_1_, *p*_2_, …, *p*_*m*−1_, 1), respectively. Again, we divided each sample size, *n*, to five intervals *m* = 5. The progressive type I interval censoring data is then generated by setting *D*_0_ = 0 and *R*_0_ = 0 and for *i* = 1, 2, …, *m*. where

- $R_{i}|R_{i-1},\ldots ,R_{0},D_{i-1},\ldots ,D_{0}∼\text{rbinom}\left(n-\sum_{i=1}^{j-1}\left(D_{j}+R_{j}\right),\frac{\left(F_{i}-F_{i-1}\right)}{\left(1-F_{i-1}\right)}\right)$.
- $D_{i}|R_{i},\ldots ,R_{0},D_{i-1},\ldots ,D_{0}=\text{floor}\left(p_{i}\times n-\sum_{j=1}^{i}D_{j}-\sum_{j=1}^{i-1}R_{j}\right)$.

where, *rbinom*(*n*, *p*) generates a random variable from the binomial distribution with parameters *n* and *p*, floor() denotes the largest integer not greater than the argument and 0 ≤ *p*_*i*_ ≤ 1, *i* = 1, 2, …, *m* − 1, *p*_*m*_ = 1 and *F* is given by (1). The following two schemes are used in progressive type-I interval censoring

- scheme 1: *p*_(1)_ = (0, 0.2, 0.50, 0.75, 1),
- scheme 2: *p*_(2)_ = (0, 0.4, 0.7, 0.9, 1).

In simulation study, both informative and non-informative priors have been used. In case of informative prior, we choose prior *θ* ∼ *gamma*(10, 10), *β* ∼ *gamma*(0.5, 10) and λ ∼ *gamma*(0.1, 10), while for non-informative prior, we choose uniform distribution *ϕ* ∼ *U*(0, 5). We run 1000 iterations for each of the two schemes. In Tables 2 and 3, we report two schemes: scheme 1, with *p*_(1)_ = (0, 0.2, 0.50, 0.75, 1) and scheme 2, with *p*_(2)_ = (0, 0.4, 0.7, 0.9, 1) respectively. For each censoring scheme, we compute the estimated mean (Mean), bias, lower and upper confidence (credible) limits for both classical and Bayesian estimates under the SEL and BSEL functions using both informative and non-informative priors. We have also presented the estimates of the posterior predictive values of the censored observation and intervals based on one and two sample predictive intervals for both schemes using both informative and non-informative priors in Table 4.

## 7 Optimal censoring scheme

Among all censoring schemes, the optimal censoring scheme provides the maximum information of the unknown parameters. The literature on optimal progressive type I Interval Censoring scheme is rather limited. [13] provided optimal progressive type I interval censoring schemes using A- and D-optimal design criteria. [12] studied optimum reliability sampling plans under progressive type I interval censoring scheme. [36] presented a method on inspection times and optimal censoring for Burr XII distribution under progressive type I interval censoring scheme. Recently, [21] presented a method on optimal censoring for inverse Weibull distribution under progressive type I interval censoring scheme and references cited there-in. In this paper, we consider the optimal censoring scheme with respect to minimum trace criteria based on [37, 38], see also [39]. For illustrative purposes, we provide a small table indicating the optimal censoring scheme with respect to minimum trace criteria. Consider the *p*^*th*^ quantile of the Dagum distribution:

$$\begin{array}{c} t_{p}={\left((p^{\frac{-1}{\beta }}-1)/\lambda \right)}^{\frac{-1}{\theta }}, \end{array}$$ (39)

Using the idea of [40], the following information measure for a given censoring scheme {*t*_1_, …, *t*_*m*_} has been used

$$\begin{array}{c} I_{w}\left(t_{1},\cdots ,t_{m}\right)=\int_{0}^{1}V{\left(t_{1},\cdots ,t_{m}\right)}_{p}w\left(p\right)dp, \end{array}$$ (40)

where, *V*(*t*_1_, …, *t*_*m*_)_*p*_ denotes the asymptotic variance of ${\hat{t}}_{p}$ the *MLE* of *t*_*p*_, based on the censoring scheme (*t*_1_, …, *t*_*m*_). Moreover, *w*(.)is a non-negative weight function such that $\int_{0}^{1}w\left(p\right)dp=1$. In this case, *V*(*t*_1_, …, *t*_*m*_)_*p*_ can be expressed as

$$\begin{array}{c} \begin{array}{c} \left[\begin{array}{ccc} \frac{\partial t_{p}}{\partial \theta } & \frac{\partial t_{p}}{\partial \beta } & \frac{\partial t_{p}}{\partial \lambda } \end{array}]I^{-1}\left(\phi \right)[\begin{array}{c} \frac{\partial t_{p}}{\partial \theta }\\ \frac{\partial t_{p}}{\partial \beta }\\ \frac{\partial t_{p}}{\partial \lambda } \end{array}]=t_{p}^{2}[\begin{array}{ccc} \frac{1}{\theta \lambda } & \frac{\log(p)}{\theta \beta^{2}(p^{\frac{1}{\beta }}-1)} & \frac{\log(t_{p})}{\theta^{2}} \end{array}]I^{-1}\left(\phi \right)[\begin{array}{c} \frac{1}{\theta \lambda }\\ \frac{\log(p)}{\theta \beta^{2}(p^{\frac{1}{\beta }}-1)}\\ \frac{\log(t_{p})}{\theta^{2}} \end{array}\right] \end{array} \end{array}$$

where *t*_*p*_ is as given by (39) and *I*^−1^(*ϕ*) is the asymptotic variance covariance matrix of the MLEs of (*θ*, *β*, λ) is given by (17) (see in Appendix).

## 8 Applications

The data sets illustrate the potentiality of the Dagum model for UK Quarterly Gas Consumption (1960–1986). The data correspond to the quarterly UK gas consumption from the first quarter of 1960 to the last quarter of 1986. The data in Table 1 are reported in [41]

**Table 1:** Real data.

year	Qtr1	Qtr2	Qtr3	Qtr4
1960	160.1	129.7	84.8	120.1
1961	160.1	124.9	84.8	116.9
1962	169.7	140.9	89.7	132.3
1963	187.3	144.1	92.9	120.1
1964	176.1	147.3	89.7	123.3
1965	185.7	155.3	99.3	131.3
1966	200.1	161.7	102.5	136.1
1967	204.9	176.1	112.1	140.9
1968	227.3	195.3	115.3	142.5
1969	244.9	214.5	118.5	267.3
1970	244.9	216.1	136.1	336.2
1971	301.0	196.9	136.1	267.3
1972	317.4	230.5	152.1	336.2
1973	371.4	240.1	158.5	355.4
1974	449.9	286.6	179.3	403.4
1975	491.5	321.8	177.7	483.5
1976	593.9	329.8	176.1	483.5
1977	584.3	395.4	187.3	485.1
1978	669.2	421.0	216.1	509.1
1979	827.7	467.5	209.7	542.7
1980	840.5	414.6	217.7	670.8
1981	848.5	437.0	209.7	701.2
1982	925.3	443.4	214.5	683.6
1983	917.3	515.5	224.1	694.8
1984	989.4	477.1	233.7	730.0
1985	1087.0	534.7	281.8	787.6
1986	1163.9	613.1	347.4	782.8

## 9 Results and discussion

The parameters of the Dagum distribution are calculated using MLE and the Bayesian approaches using both SEL and BSEL function with informative and non-informative prior for *n* = 50 and *n* = 100. Table 2 gives the values of the parameters when *w* = 0.3 and the proportions are *p*_(1)_ = (0, 0.4, 0.7, 0.9, 1). Table 3 gives the values of the parameters when *w* = 0.3 and the proportions are *p*_(2)_ = (0, 0.2, 0.5, 0.75, 1). Table 4 gives the values of predicted values *Y*_(*s*)_ for one and two samples schemes using proportion *p*_(1)_ = (0, 0.4, 0.7, 0.9, 1) and *p*_(2)_ = (0, 0.2, 0.50, 0.75, 1), for sample sizes n = 50 and n = 100. We observe from Tables 2 & 3, in case of MLE, the MSEs of the two censoring percentage, *p*_(1)_ and *p*_(2)_ are the same for the scale parameter λ while for the two shape parameters *θ* and *β* are different. It is also observe that the Bayes estimates based on SEL and BSEL performs better than maximum likelihood estimates in terms of bias and MSEs. Also, in all the cases, MSEs decrease as we increase the sample sizes. It verifies the consistency of the estimators. The results also show that the performance of SEL based on informative prior is more or less same as non-informative prior both in terms of bias and MSEs values. However, in terms of MSEs of the parameters *θ* and λ in case of censoring percentage, *p*_(1)_ performs better than *p*_(2)_ while for the parameter *β*, *p*_(2)_ performs better than *p*_(1)_. Further, we observe that the performance of BSEL for both informative and non-informative priors are not better off than the SEL. It is to be noted that under non-informative prior based on BSEL(BSEL-non) function, MSEs in *p*_(2)_ performs better than *p*_(1)_ for all parameter values *θ*, *β* and λ. The performance of the Bayes estimates under SEL improve in almost all cases with the increase in the sample size *n*. This behaviour holds true under both the censoring schemes and different choices of *n*. Further, we observe that as the sample size gets larger, average asymptotic confidence interval estimates and credible interval estimates decreases for the parameters *β* and λ. Finally, it is seen that the credible intervals have smaller interval lengths. From Table 4, we observe that predictive estimates based on SEL with informative prior performs better than non-informative prior for both one and two sample schemes. We note that the censoring percentage in *p*_(1)_ is more than *p*_(2)_, so the experiment rapidly gets finished in case of *p*_(1)_ than *p*_(2)_, thus censoring percentage *p*_(1)_ saves the time and money more than *p*_(2)_. In Table 5, we report the decision variables *D*_0_ and *p*_0_ and different values of *m* for optimal censoring scheme. From Table 5, we observe that the optimal values of *D*_0_ decreases as the number of inspection times *m* increases which implies that as the number of inspection time increases, intermediate time between two successive inspections get shorter. Again, with the increase of number of inspections, the optimal values increase. for real data analysis the results are presented in Table 6.

**Table 2:** Maximum likelihood and Bayes estimates based on scheme 1, *w* = 0.3, *m* = 5, *p*_(1)_ = (0, 0.4, 0.7, 0.9, 1).

	n = 50					n = 100				
Parameters	Mean	bias	MSE	lower	upper	Mean	bias	MSE	LCL	UCL
	MLE					MLE				
*θ*	0.9708	1.0292	1.2673	0.0264	1.0354	0.0261	0.6104	0.4950	2.0349	3.1859
*β*	0.1184	0.1575	0.0253	0.0264	0.0354	0.0186	0.14029	0.0896	1.4248	2.2946
λ	0.0068	0.9999	0.9999	0.0181	0.0188	2.437e-06	0.9999	0.9999	0.000	2.6839e-05
	SEL-in					SEL-in				
*θ*	1.8996	0.1004	0.0098	1.8990	1.9014	1.90111	0.0988	0.0098	1.8991	1.9007
*β*	2.0996	0.0996	0.0101	2.0909	2.1012	2.09809	0.0980	0.0096	2.0998	2.1026
λ	0.8982	0.1018	0.0099	0.8096	0.9011	0.90052	0.0995	0.0098	0.8996	0.90111
	SEL-non					SEL-non				
*θ*	1.8995	0.1004	0.0098	1.8091	1.9005	1.90111	0.0988	0.0098	1.8998	1.9019
*β*	2.1001	0.1001	0.0101	2.0088	2.1011	2.09809	0.0980	0.0096	2.0968	2.0997
λ	0.5389	0.4610	0.0099	0.8199	0.9020	0.90052	0.0995	0.0098	0.8998	0.9011
	BSEL-in					BSEL-in				
*θ*	2.2268	0.2268	0.0515	1.8990	1.9116	2.1848	0.1848	0.0886	2.0054	2.3482
*β*	2.0063	0.0063	4.04e-05	2.1648	2.1889	2.0027	0.0027	0.0913	1.9876	2.1437
λ	0.5402	0.4598	0.2114	0.9298	0.9878	0.5403	0.4597	0.05938	0.4837	0.8192
	BSEL-non					BSEL-non				
*θ*	1.8995	0.10046	0.05111	1.8986	1.9005	2.1848	0.1848	0.0886	2.0054	2.3482
*β*	2.1002	0.1002	3.67e-05	2.0988	2.1011	2.0027	0.0027	0.0913	1.9876	2.1437
λ	0.9009461	0.0991	0.21108	0.8995	0.9020	0.5403	0.4597	0.05938	0.4837	0.8192

**Table 3:** Maximum likelihood and Bayes estimates based on scheme 2, *w* = 0.3, *m* = 5, *p*_(2)_ = (0, 0.2, 0.50, 0.75, 1).

	n = 50					n = 100				
Parameters	Mean	bias	MSE	lower	upper	Mean	bias	MSE	LCL	UCL
	MLE					MLE				
*θ*	0.0275	0.7493	1.2776	1.3572	4.1413	0.02610	0.6104	0.4950	2.03499	3.18587
*β*	0.01865	0.1352	0.2692	1.0409	2.6887	0.01859	0.1403	0.0895	1.42480	2.29461
λ	1.338e-06	0.9999	0.9999	0.0000	2.440e-05	2.43e-06	0.9999	0.9999	0.0000	2.683e-05
	SEL-in					SEL-in				
*θ*	1.9009	0.0098	0.0245	1.8999	1.90143	1.9001	0.0999	0.0099	1.89906	1.90072
*β*	2.1007	0.01015	0.0009	2.0998	2.10125	2.10082	0.10082	0.01017	2.09949	2.10239
λ	0.9004	0.0099	0.1367	0.8996	0.90115	0.9002	0.09981	0.0099	0.89938	0.90074
	SEL-non					SEL-non				
*θ*	1.8995	0.1005	0.0101	1.8985	1.9005	1.90111	0.09888	0.00978	1.89976	1.90187
*β*	2.1002	0.10019	0.01004	2.0988	2.1011	2.09809	0.09809	0.009621	2.09677	2.09973
λ	0.9009	0.09905	0.0098	0.8995	0.9020	0.90052	0.99479	0.00989	0.89976	0.90109
	BSEL-in					BSEL-in				
*θ*	2.15542	0.1554	0.0242	2.0648	2.3981	2.18426	0.18426	0.08792	2.15394	2.26975
*β*	2.02995	0.02995	0.0009	1.9983	2.3698	2.00438	0.00438	0.00093	2.02948	2.08792
λ	0.6303	0.3697	0.1367	0.6099	0.6919	0.54011	0.45989	0.00682	0.53898	0.55993
	BSEL-non					BSEL-non				
*θ*	2.1545	0.1545	0.0238	1.91985	1.9200	2.18484	0.18484	0.09979	2.14391	2.26975
*β*	2.02957	0.0296	0.0009	2.0388	2.3941	2.00273	0.00273	0.00693	2.00182	2.08792
λ	0.63066	0.36933	0.1364	0.6195	0.9002	0.54031	0.45969	0.00982	0.53698	0.55192

**Table 4:** Predictive estimates based on scheme 1 and scheme 2, *m* = 5, *p*_(1)_ = (0, 0.4, 0.7, 0.9, 1), *p*_(2)_ = (0, 0.2, 0.50, 0.75, 1).

n	Type		Mean	bias	MSE	LCL	UCL
50, *p*_1_			SEL_non				
	one sample	*Y*_*s*_	1.1991911	0.800808	0.6412950	1.1984408	1.199796
	two samples	*Y*_*s*_	0.9005342	0.896534	0.0803773	0.8999234	0.901019
			SEL_in				
	one sample	*Y*_*s*_	1.2010362	0.798963	0.638343	1.1998073	1.201669
	two samples	*Y*_*s*_	0.0031222	1.314e-06	0.000877	0.0017258	0.004087
100, *p*_1_			SEL_non				
	one sample	*Y*_*s*_	1.2008427	0.799157	0.6386526	1.1999928	1.201471
	two samples	*Y*_*s*_	0.9000144	0.896014	0.0802841	0.8994546	0.900604
			SEL_in				
	one sample	*Y*_*s*_	1.20083481	0.799165	0.6386655	1.1997385	1.202074
	two samples	*Y*_*s*_	0.00434036	0.000340	6.094e–07	0.0026393	0.005397
50, *p*_2_			SEL_non				
	one sample	*Y*_*s*_	1.19994440	0.800055	0.640089	1.198668	1.201423
	two samples	*Y*_*s*_	0.90062120	0.896621	0.803930	0.899512	0.901870
			SEL_in				
	one sample	*Y*_*s*_	1.20125620	0.798743	0.637992	1.199918	1.202989
	two samples	*Y*_*s*_	0.00452716	0.000527	6.191e-07	0.003342	0.005583
100, *p*_2_			SEL_non				
	one sample	*Y*_*s*_	1.2000322	0.799967	0.6399486	1.199348	1.200539
	two samples	*Y*_*s*_	0.9000486	0.896048	0.8029033	0.898844	0.900711
			SEL_in				
	one sample	*Y*_*s*_	1.200673316	0.799326	0.6389233	1.199747	1.201356
	two samples	*Y*_*s*_	0.004647491	0.000647	6.355e-07	0.003626	0.005362

**Table 5:** Optimal censoring scheme for trace-optimal criteria when *θ* = 2, *β* = 3, λ = 2 and *n* = 50, 100 and different values of *m*.

n	m	D_0_, *p*_0_	trace-optimal times
50	2	(0.67,0.00)	(0.54,1.98)
	3	(0.54,0.00)	(0.43,1.73,2.26)
	4	(0.49,0.00)	(0.38,1.49,2.1,1,2.82)
	5	(0.37,0.00)	(0.32,0.96,1.74,2.51,2.97)
100	5	(0.46,0.00)	(0.37,1.06,1.89,2.74,2.99)
	6	(0.42,0.00)	(0.34,1.01,1.72, 2.15,2.27,2.45)
	7	(0.39,0.00)	(0.29,0.94,1.63,1.97,2.15,2.28,2.31)
	8	(0.34,0.00)	(0.24,0.78,1.43,1.82,1.90,1.97,2.16,2.22)
	9	(0.31,0.00)	(0.21,0.34,0.68,0.89,1.14,1.27,1.45,1.51,1.67)
	10	(0.29,0.00	(0.18,0.23,0.35,0.39,0.47,0.49,0.61,0.70,0.83,0.89)

**Table 6:** Optimal censoring scheme for trace-optimal criteria when *θ* = 3.2, *β* = 5.9, λ = 1.75, *n* = 88 using real data.

m	D_0_, *p*_0_	trace-optimal times
2	(414.6,0.00)	(317.4, 670.8)
3	(371.3,0.00)	(286.6, 329.6,483.5)
4	(336.3,0.00)	(241.1,321.8,437.0,467.5)

In real data analysis, we could not observe any changes in the results for both the percentage censoring (i.e., *p*_(1)_ and *p*_(2)_), thus we just reported one of them (i.e., *p*_(2)_). In addition, there is no difference between SEL results and BSEL as presented in Table 7. In the next Tables we denote lower confidence limit (LCL) and upper confidence limit (UCL).

**Table 7:** Maximum likelihood and Bayes estimates based on scheme 2, m = 4, *p*_(2)_ = (0, 0.2, 0.50, 0.75, 1), *θ* = 3.2, *β* = 5.9, λ = 1.75 using real data.

Parameters	Mean	standard error	LCL	UCL
MLE				
*θ*	4.490103	1.290103	4.490046	4.490159
*β*	5.984063	0.084063	5.983605	5.984521
λ	1.635266	0.114734	1.635237	1.635296
SEL_in				
*θ*	3.098201	0.101799	3.097116	3.099765
*β*	6.000055	0.031639	5.999388	6.000530
λ	1.649941	0.031639	0.000000	0.000011
*Y*_*s*_	0.002201	0.00199	0.001116	0.003769
SEL_non				
*θ*	3.1012967	0.098707	3.099783	3.102186
*β*	5.9991934	0.099192	5.998141	6.000093
λ	1.6507618	0.099192	1.649200	1.651973
*Y*_*s*_	0.9012967	0.099241	0.899783	0.902186

## 10 Conclusions

In this paper we discussed parameter estimation for the Dagum distribution based on progressive type-I interval censored data. Under classical set up we obtain maximum likelihood estimates and confidence intervals. In Bayesian framework, we have obtained Bayes estimates under SEL and BSEL functions using independent informative gamma and non informative uniform priors for both scale and two shape parameters. Moreover, Bayes predictive estimates and intervals are obtained using one and two sample schemes. Further, the optimal censoring scheme based on minimum trace criteria is discussed. Finding an optimum censoring scheme is an open problem from the computational point of view. More work is needed along that direction. So, present work may help the industry people to analyze progressive type-I interval censored lifetime data with optimal censoring. A future work is to estimate procedures of stress-strength reliability for Dagum distribution. Another future work is to study and compare the Bayesian estimation based on maximum likelihood and based on maximum product of spacing to estimate the stress-strength reliability of Dagum distribution.

### Appendix

The second derivatives of the information matrix of Dagum distribution based on progressive type I interval censoring with respect to *θ*, *β* and λ are given by the following equations:

$$\begin{array}{ccc} \Psi_{\theta \theta } & = & \sum_{j=1}^{m}(\frac{D_{j}{\left(U_{j}\right)}_{\theta }^{2}}{U_{j}^{2}}+\frac{D_{j}{\left(U_{j}\right)}_{\theta \theta }}{U_{j}}-\frac{\beta \lambda R_{j}\log^{2}(t_{j})((\beta \lambda -t_{j}^{\theta }){\left(\lambda t_{j}^{-\theta }+1\right)}^{\beta }+t_{j}^{\theta })}{V_{j}^{2}}), \end{array}$$ (41)

$$\begin{array}{ccc} \Psi_{\beta \beta } & = & \sum_{j=1}^{m}(\frac{R_{j}{\left(V_{j}\right)}_{\beta \beta }(t_{j}^{\theta }+\lambda )}{V_{j}^{2}}-\frac{D_{j}{\left(U_{j}\right)}_{\beta }^{2}{\left(\lambda t_{j-1}^{-\theta }+1\right)}^{2\beta }{\left(\lambda t_{j}^{-\theta }+1\right)}^{2\beta }}{U_{j}^{2}}\\ & & -\frac{D_{j}{\left(U_{j}\right)}_{\beta }{\left(\lambda t_{j-1}^{-\theta }+1\right)}^{\beta }{\left(\lambda t_{j}^{-\theta }+1\right)}^{\beta }}{U_{j}}), \end{array}$$ (42)

$$\Psi_{\lambda \lambda }=\sum_{j=1}^{m}(\frac{D_{j}{\left(U_{j}\right)}_{\lambda \lambda }}{U_{j}}-\frac{D_{j}{\left(U_{j}\right)}_{\lambda }^{2}}{U_{j}^{2}}+\frac{\beta R_{j}{\left(V_{j}\right)}_{\beta }}{V_{j}^{2}}),$$ (43)

$$\begin{array}{ccc} \Psi_{\theta \beta } & = & \sum_{j=1}^{m}(\frac{\beta \lambda R_{j}\log\left(t_{j}\right){\left(V_{j}\right)}_{\beta }}{V_{j}^{2}}+\frac{\lambda R_{j}\log\left(t_{j}\right)t_{j}^{-\theta }{\left(1+\lambda t_{j}^{-\theta }\right)}^{-\beta -1}{\left(V_{j}\right)}_{\theta \lambda }}{\beta \left(1+\beta \right)V_{j}}\\ & & +\frac{D_{j}{\left(U_{j}\right)}_{\theta \beta }}{U_{j}}-\frac{D_{j}{\left(U_{j}\right)}_{\beta }{\left(U_{j}\right)}_{\theta }}{U_{j}^{2}}), \end{array}$$ (44)

$$\begin{array}{ccc} \Psi_{\theta \lambda } & = & \sum_{j=1}^{m}(\frac{\beta^{2}\lambda R_{j}\log(t_{j})}{V_{j}^{2}}+\frac{\beta R_{j}\log(t_{j})(\beta \lambda -t_{j}^{\theta })}{V_{j}(t_{j}^{\theta }+\lambda )}-\frac{D_{j}{\left(U_{j}\right)}_{\theta }{\left(U_{j}\right)}_{\lambda }}{U_{j}^{2}}\\ & & +\frac{D_{j}{\left(U_{j}\right)}_{\theta \lambda }}{U_{j}}), \end{array}$$ (45)

$$\begin{array}{ccc} \Psi_{\beta \lambda } & = & \sum_{j=1}^{m}(\frac{R_{j}}{V_{j}}-\frac{\beta R_{j}{\left(V_{j}\right)}_{\beta }}{V_{j}^{2}{\left(t_{j}^{\theta }+\lambda \right)}^{2}}-\frac{D_{j}{\left(U_{j}\right)}_{\beta }{\left(U_{j}\right)}_{\lambda }}{U_{j}^{2}}+\frac{D_{j}{\left(U_{j}\right)}_{\beta \lambda }}{U_{j}}), \end{array}$$ (46)

where

$$\begin{array}{ccc} {\left(U_{j}\right)}_{\theta \theta } & \equiv & \frac{\partial U_{j}(\theta ,\beta ,\lambda )}{\partial \theta^{2}}=\beta \lambda (\frac{\log^{2}(t_{j-1}){\left(\lambda t_{j-1}^{-\theta }+1\right)}^{-\beta }(t_{j-1}^{\theta }-\beta \lambda )}{{\left(t_{j-1}^{\theta }+\lambda \right)}^{2}}\\ & & -\frac{\log^{2}(t_{j}){\left(\lambda t_{j}^{-\theta }+1\right)}^{-\beta }(t_{j}^{\theta }-\beta \lambda )}{{\left(t_{j}^{\theta }+\lambda \right)}^{2}}), \end{array}$$

$$\begin{array}{ccc} {\left(U_{j}\right)}_{\theta \lambda } & \equiv & \frac{\partial U_{j}(\theta ,\beta ,\lambda )}{\partial \theta \lambda }=\beta (\frac{\log(t_{j}){\left(\lambda t_{j}^{-\theta }+1\right)}^{-\beta }(t_{j}^{\theta }-\beta \lambda )}{{\left(t_{j}^{\theta }+\lambda \right)}^{2}}\\ & & -\frac{\log(t_{j-1}){\left(\lambda t_{j-1}^{-\theta }+1\right)}^{-\beta }(t_{j-1}^{\theta }-\beta \lambda )}{{\left(t_{j-1}^{\theta }+\lambda \right)}^{2}}), \end{array}$$

$$\begin{array}{ccc} {\left(U_{j}\right)}_{\beta \lambda } & \equiv & \frac{\partial U_{j}(\theta ,\beta ,\lambda )}{\partial \beta \lambda }=t_{j-1}^{-\theta }{\left(\lambda t_{j-1}^{-\theta }+1\right)}^{-\beta -1}(1-\beta \log(\lambda t_{j-1}^{-\theta }+1))+t_{j}^{-\theta }\\ & & \times {\left(\lambda t_{j}^{-\theta }+1\right)}^{-\beta -1}(\beta \log(\lambda t_{j}^{-\theta }+1)-1), \end{array}$$

$$\begin{array}{ccc} {\left(V_{j}\right)}_{\theta \beta } & \equiv & \frac{\partial U_{j}(\theta ,\beta ,\lambda )}{\partial \theta \beta }=-\log(t_{j}){\left(\lambda t_{j}^{-\theta }+1\right)}^{\beta }(\beta \lambda \log(\lambda t_{j}^{-\theta }+1)\\ & & +t_{j}^{\theta }(-\log(\lambda t_{j}^{-\theta }+1))+\lambda ), \end{array}$$

$$\begin{array}{c} {\left(V_{j}\right)}_{\beta \beta }\equiv \frac{\partial U_{j}(\theta ,\beta ,\lambda )}{\partial \beta^{2}}=t_{j}^{-\theta }{\left(\lambda t_{j}^{-\theta }+1\right)}^{\beta +1}\log^{2}(\lambda t_{j}^{-\theta }+1), \end{array}$$

$$\begin{array}{c} {\left(V_{j}\right)}_{\beta }=t_{j}^{-\theta }{\left(\lambda t_{j}^{-\theta }+1\right)}^{\beta +1}\log(\lambda t_{j}^{-\theta }+1), \end{array}$$

$$\begin{array}{c} {\left(U_{j}\right)}_{\lambda \lambda }\equiv \frac{\partial U_{j}(\theta ,\beta ,\lambda )}{\partial \lambda^{2}}=\beta \left(\beta +1\right)(\frac{{\left(\lambda t_{j}^{-\theta }+1\right)}^{-\beta }}{{\left(t_{j}^{\theta }+\lambda \right)}^{2}}-\frac{{\left(\lambda t_{j-1}^{-\theta }+1\right)}^{-\beta }}{{\left(t_{j-1}^{\theta }+\lambda \right)}^{2}}), \end{array}$$

and *U*_*j*_, *V*_*j*_, (*U*_*j*_)_*θ*_, (*U*_*j*_)_*β*_, (*U*_*j*_)_λ_ are given by (11)–(15), respectively.
